# Dynamic trajectories of serum sodium and urine output and their association with prognosis in sepsis: a group-based multi-trajectory modelling analysis using MIMIC-IV

**DOI:** 10.7189/jogh.16.04308

**Published:** 2026-09-04

**Authors:** Xiuming Zhuo, Xiaolong Xu, Hongyi Chen

**Affiliations:** Shengli Clinical Medical College of Fujian Medical University; Department of Emergency, Fuzhou University Affiliated Provincial Hospital; Fujian Provincial Key Laboratory of Emergency Medicine, Fuzhou City, Fujian Province, China

**Keywords:** serum sodium, urine output, sepsis, prognosis, MIMIC-IV

## Abstract

**Background:**

Serum sodium and urine output (UO) are routinely monitored indicators of electrolyte homeostasis, renal perfusion, and organ function in sepsis. However, the prognostic relevance of their combined longitudinal patterns remains unclear.

**Methods:**

We conducted a retrospective cohort study using the Medical Information Mart for Intensive Care IV database. Group-based multi-trajectory modelling identified combined trajectories of serum sodium and UO across four 24-hour periods during the first 96 hours after intensive care unit admission. Cox regression evaluated associations between trajectory subtypes and 30-day mortality, with subgroup and sensitivity analyses.

**Results:**

Among 7127 patients with sepsis, five trajectory subtypes were identified. Subtype 1 was characterised by a mild initial decrease in serum sodium followed by recovery and gradually increasing UO. Compared with subtype 1, subtype 2, characterised by mildly increasing serum sodium and gradually decreasing UO, showed the highest 30-day mortality risk in the fully adjusted model (HR = 1.85; 95% CI = 1.56–2.19, *P* < 0.001). Higher 30-day mortality was also observed for subtype 3, characterised by relatively stable serum sodium and UO that increased and then decreased (HR = 1.22; 95% CI = 1.03–1.46, *P* = 0.026); subtype 4, characterised by gradually increasing serum sodium and UO that increased before levelling off (HR = 1.31; 95% CI = 1.10–1.56, *P* = 0.003); and subtype 5, characterised by gradually decreasing serum sodium and UO that increased before levelling off (HR = 1.53; 95% CI = 1.29–1.82, *P* < 0.001). Subgroup and sensitivity analyses generally supported these findings.

**Conclusions:**

Five combined serum sodium and UO trajectory subtypes were identified in sepsis. Subtype 1 was associated with a favourable prognosis, whereas subtypes 2 and 5 carried the highest mortality risks. Early combined trajectories of serum sodium and UO may support prognostic risk stratification, although causal inferences cannot be drawn from this observational study.

Sepsis is life-threatening organ dysfunction caused by a dysregulated host response to infection, which can further progress to multiple organ dysfunction and even death [1]. In 2021, an estimated 166 million sepsis cases and 21.4 million related deaths occurred worldwide, accounting for 31.5% of global mortality [2]. Sepsis patients frequently develop comorbidities, particularly electrolyte disturbances and acute kidney injury (AKI). The electrolyte status in sepsis is often dynamically imbalanced and strongly linked to patient outcomes [3]. Concurrently, sepsis patients are at high risk for AKI, which further elevates mortality. A retrospective study indicates an AKI incidence of 33.5% among sepsis patients, with an excess attributable mortality of 58.6% [4]. Therefore, timely detection and management of electrolyte disorders and renal function changes are crucial for improving clinical outcomes.

Serum sodium, the most abundant extracellular cation, plays a key role in maintaining osmotic pressure and blood volume stability, serving as a pivotal indicator of electrolyte status [5]. Urine output (UO) is regarded as a real-time window reflecting renal perfusion and filtration function [6]. Growing evidence suggests that both serum sodium and UO levels correlate with sepsis prognosis. In one retrospective study, peak serum sodium (154.9 mmol/L) predicted 28-day mortality in elderly sepsis patients, with an area under the curve of 0.753 [7]. Urine output inversely correlates with mortality; every 50 mL increase in 24-hour UO corresponded to a 1% reduction in mortality risk [8]. However, patient conditions evolve dynamically, especially within the intensive care unit (ICU), where physiological parameters and organ functions continuously change [9]. Therefore, investigating the link between dynamic changes in serum sodium/UO and sepsis prognosis is necessary. Recent methodological advancements, such as group-based multi-trajectory modelling (GBMTM), provide a powerful tool for this purpose. Group-based multi-trajectory modelling can identify latent subtypes with unique evolution patterns from repeated measures of multiple variables, revealing clinically significant dynamic patterns obscured by overall average trends [10].

Consequently, this study aimed to analyse the dynamic trajectories of serum sodium and UO in sepsis patients using GBMTM. We sought to identify trajectory subtypes with distinct characteristics and explore their association with 30-day mortality. Given that serum sodium and UO reflect electrolyte homeostasis and effective renal perfusion status respectively, the core hypotheses of this study are as follows:

• heterogeneous combined dynamic trajectories of serum sodium and UO exist among patients with sepsis;

• specific trajectories indicative of sustained deterioration in renal perfusion (persistently decreased UO) or internal environmental imbalance (serum sodium persistently deviating from the normal reference range) may still correlate with elevated mortality risk after adjustment for baseline disease severity;

• clinical conditions or therapeutic backgrounds including body mass index (BMI) and renal replacement therapy (RRT) may modify the association between trajectory subtypes and mortality risk.

## METHODS

### Study population

Data were sourced from the Medical Information Mart for Intensive Care IV (MIMIC-IV), version 2.2 database, containing clinical data from approximately 60,000 ICU patients at Beth Israel Deaconess Medical Center (2008–2019). All data were de-identified, thus eliminating the need for additional ethical approval.

The study initially included 25,596 patients with sepsis. Exclusion criteria were:

• ICU stay <96 hours (n = 15,391);

• age <18 years (n = 0);

• non-first ICU admission for patients with multiple hospitalisations (n = 3,078).

**Figure 1:**
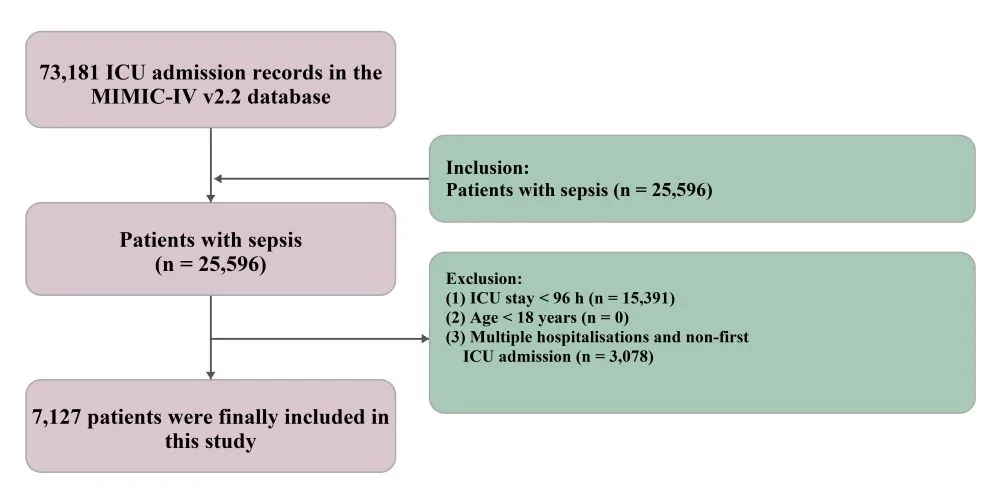
Flowchart of patient selection. ICU – intensive care unit, MIMIC-IV – medical information mart for intensive care IV.

Finally, 7,127 patients were included (**Figure 1**).

### Disease definition

Patients with sepsis were diagnosed in accordance with the Third International Consensus Definitions for Sepsis and Septic Shock (Sepsis-3). Sepsis is defined as life-threatening organ dysfunction resulting from a dysregulated host response to infection. Clinically, organ dysfunction is generally identified by an increase of ≥2 points in the Sequential Organ Failure Assessment (SOFA) score relative to the baseline value, accompanied by confirmed or highly suspected infection [11].

### Variables

#### Exposure variables

Exposure variables were serum sodium and UO. The observation window covered the first 96 hours after ICU admission, which was divided into four-time intervals: 0–24 hours, 24–48 hours, 48–72 hours, and 72–96 hours. For the primary analysis, if multiple measurements were recorded within a single time interval, the minimum serum sodium value and minimum UO value in that interval were extracted to represent the worst electrolyte status and UO level during the corresponding period. Meanwhile, the mean values of serum sodium and UO within each time interval were collected for subsequent sensitivity analyses of exposure variable definitions.

#### Outcome variables

The primary outcome was 30-day mortality after ICU admission. Secondary outcomes included 90-day mortality and in-hospital mortality.

### Covariates

Covariates included demographic information, vital signs, laboratory results, severity scores, treatment details, and comorbidities (**Table 1**).

**Table 1:** Baseline characteristics of included patients at first ICU admission*

Characteristics	Total	Subtype 1	Subtype 2	Subtype 3	Subtype 4	Subtype 5	*P*-value
	**(n = 7,127)**	**(n = 1,417)**	**(n = 1,366)**	**(n = 1,508)**	**(n = 1,441)**	**(n = 1,395)**	
Sex							0.230
*Female*	3,082 (43.2)	619 (43.7)	622 (45.5)	641 (42.5)	595 (41.3)	605 (43.4)	
*Male*	4,045 (56.8)	798 (56.3)	744 (54.5)	867 (57.5)	846 (58.7)	790 (56.6)	
Age, in years	65.42 (16.61)	64.74 (17.11)	64.79 (17.08)	65.90 (16.26)	65.25 (15.96)	66.39 (16.62)	0.033
Race							0.091
*White*	4,503 (63.2)	882 (62.2)	853 (62.4)	967 (64.1)	927 (64.3)	874 (62.7)	
*Black*	530 (7.4)	113 (8.0)	114 (8.3)	89 (5.9)	92 (6.4)	122 (8.7)	
*Other race*	2,094 (29.4)	422 (29.8)	399 (29.2)	452 (30.0)	422 (29.3)	399 (28.6)	
Sepsis onset period							<0.001
*Post-ICU*	4,208 (59.0)	842 (59.4)	793 (58.1)	953 (63.2)	775 (53.8)	845 (60.6)	
*Pre-ICU*	2,919 (41.0)	575 (40.6)	573 (41.9)	555 (36.8)	666 (46.2)	550 (39.4)	
BMI (kg/m^2^)	29.37 (8.21)	29.36 (8.08)	28.88 (7.89)	29.55 (8.42)	29.95 (8.19)	29.06 (8.38)	0.005
Heart rate (times/min)	88.05 (16.94)	87.93 (17.30)	89.12 (17.11)	87.61 (16.73)	87.58 (16.53)	88.10 (17.04)	0.105
SpO_2_	97.06 (2.30)	97.17 (2.23)	96.75 (2.43)	97.29 (2.01)	97.07 (2.12)	96.99 (2.64)	<0.001
Anion gap (mmol/L)	17.30 (5.13)	17.31 (5.26)	17.34 (4.88)	16.73 (5.05)	17.38 (4.87)	17.76 (5.51)	<0.001
Chloride (mmol/L)	106.47 (6.88)	107.01 (6.23)	105.06 (6.59)	106.89 (6.09)	104.07 (6.71)	109.33 (7.53)	<0.001
Platelet (K/μL)	164.00 (111.00, 229.50)	170.00 (113.00, 226.00)	169.50 (114.00, 240.00)	165.00 (114.00, 231.00)	167.00 (111.00, 231.00)	153.00 (102.00, 220.00)	<0.001
Potassium (mmol/L)	4.65 (0.86)	4.65 (0.88)	4.62 (0.88)	4.65 (0.83)	4.66 (0.87)	4.67 (0.87)	0.683
pH (units)	7.30 (0.12)	7.29 (0.12)	7.31 (0.12)	7.30 (0.11)	7.31 (0.11)	7.28 (0.13)	<0.001
Baseline eGFR	68.47 (33.81)	69.59 (33.51)	69.46 (34.87)	70.69 (33.43)	66.85 (33.43)	65.64 (33.65)	<0.001
Fluid balance (mL)	6,734.06 (2,927.85, 12,202.20)	7,640.75 (3,786.10, 12,705.67)	4,926.65 (1,641.88, 10,357.73)	7,251.56 (3,413.70, 12,415.12)	5,945.32 (2,574.75, 10,852.38)	7,850.10 (3,460.00, 13,831.68)	<0.001
Lactate (mmol/L)	2.20 (1.50, 3.80)	2.30 (1.50, 3.80)	2.10 (1.40, 3.40)	2.20 (1.40, 3.60)	2.20 (1.40, 3.50)	2.50 (1.60, 4.50)	<0.001
BUN (mg/dL)	23.00 (16.00, 39.00)	23.00 (15.00, 36.00)	23.00 (15.00, 38.00)	23.00 (16.00, 37.00)	25.00 (16.00, 43.00)	24.00 (16.00, 40.00)	<0.001
WBC (K/μL)	14.40 (10.40, 19.40)	14.80 (10.90, 19.90)	13.90 (9.80, 18.98)	14.50 (10.60, 19.10)	14.50 (10.40, 19.50)	14.40 (10.15, 19.85)	0.018
Calcium (mg/dL)	7.93 (0.91)	7.90 (0.89)	7.96 (0.90)	7.94 (0.85)	7.98 (0.86)	7.87 (1.04)	0.004
SAPS II	42.41 (14.52)	43.38 (15.15)	40.50 (14.37)	41.68 (13.60)	42.37 (14.08)	44.10 (15.16)	<0.001
SOFA	6.00 (4.00, 9.00)	6.00 (4.00, 9.00)	6.00 (3.00, 9.00)	6.00 (4.00, 9.00)	6.00 (4.00, 9.00)	7.00 (4.00, 10.00)	<0.001
GCS	13.11 (3.34)	12.91 (3.60)	13.17 (3.15)	13.23 (3.29)	13.07 (3.23)	13.17 (3.41)	0.085
Diuretic therapy							<0.001
*No*	3,294 (46.2)	605 (42.7)	692 (50.7)	670 (44.4)	655 (45.5)	672 (48.2)	
*Yes*	3,833 (53.8)	812 (57.3)	674 (49.3)	838 (55.6)	786 (54.5)	723 (51.8)	
Invasive ventilation							<0.001
*No*	2,143 (30.1)	351 (24.8)	564 (41.3)	416 (27.6)	415 (28.8)	397 (28.5)	
*Yes*	4,984 (69.9)	1,066 (75.2)	802 (58.7)	1,092 (72.4)	1,026 (71.2)	998 (71.5)	
β-blockers							<0.001
*No*	4,763 (66.8)	954 (67.3)	969 (70.9)	948 (62.9)	938 (65.1)	954 (68.4)	
*Yes*	2,364 (33.2)	463 (32.7)	397 (29.1)	560 (37.1)	503 (34.9)	441 (31.6)	
Vasoactive drugs							<0.001
*No*	3,595 (50.4)	702 (49.5)	779 (57.0)	715 (47.4)	786 (54.5)	613 (43.9)	
*Yes*	3,532 (49.6)	715 (50.5)	587 (43.0)	793 (52.6)	655 (45.5)	782 (56.1)	
Mannitol treatment							<0.001
*No*	6,956 (97.6)	1,392 (98.2)	1,326 (97.1)	1,481 (98.2)	1,374 (95.4)	1,383 (99.1)	
*Yes*	171 (2.4)	25 (1.8)	40 (2.9)	27 (1.8)	67 (4.6)	12 (0.9)	
RRT							<0.001
*No*	6,336 (88.9)	1,283 (90.5)	1,150 (84.2)	1,401 (92.9)	1,337 (92.8)	1,165 (83.5)	
*Yes*	791 (11.1)	134 (9.5)	216 (15.8)	107 (7.1)	104 (7.2)	230 (16.5)	
Albumin infusion							<0.001
*No*	5,180 (72.7)	1,015 (71.6)	1,048 (76.7)	1,048 (69.5)	1,106 (76.8)	963 (69.0)	
*Yes*	1,947 (27.3)	402 (28.4)	318 (23.3)	460 (30.5)	335 (23.2)	432 (31.0)	
Congestive heart failure							0.163
*No*	4,865 (68.3)	986 (69.6)	915 (67.0)	1,027 (68.1)	1,010 (70.1)	927 (66.5)	
*Yes*	2,262 (31.7)	431 (30.4)	451 (33.0)	481 (31.9)	431 (29.9)	468 (33.5)	
Chronic pulmonary disease							0.662
*No*	5,129 (72.0)	1,043 (73.6)	976 (71.4)	1,081 (71.7)	1,029 (71.4)	1,000 (71.7)	
*Yes*	1,998 (28.0)	374 (26.4)	390 (28.6)	427 (28.3)	412 (28.6)	395 (28.3)	
Renal disease							0.007
*No*	5,646 (79.2)	1,125 (79.4)	1,068 (78.2)	1,205 (79.9)	1,181 (82.0)	1,067 (76.5)	
*Yes*	1481 (20.8)	292 (20.6)	298 (21.8)	303 (20.1)	260 (18.0)	328 (23.5)	
Diabetes							0.007
*No*	5,038 (70.7)	1,012 (71.4)	1,015 (74.3)	1,030 (68.3)	1,010 (70.1)	971 (69.6)	
*Yes*	2,089 (29.3)	405 (28.6)	351 (25.7)	478 (31.7)	431 (29.9)	424 (30.4)	
Hypertension							0.009
*No*	4125 (57.9)	814 (57.4)	817 (59.8)	868 (57.6)	783 (54.3)	843 (60.4)	
*Yes*	3,002 (42.1)	603 (42.6)	549 (40.2)	640 (42.4)	658 (45.7)	552 (39.6)	
Acute liver failure							0.012
*No*	6,795 (95.3)	1,359 (95.9)	1,289 (94.4)	1,455 (96.5)	1,378 (95.6)	1,314 (94.2)	
*Yes*	332 (4.7)	58 (4.1)	77 (5.6)	53 (3.5)	63 (4.4)	81 (5.8)	
30-day mortality							<0.001
*No*	5,600 (78.6)	1,187 (83.8)	993 (72.7)	1,232 (81.7)	1,132 (78.6)	1,056 (75.7)	
*Yes*	1,527 (21.4)	230 (16.2)	373 (27.3)	276 (18.3)	309 (21.4)	339 (24.3)	
90-day mortality							<0.001
*No*	5,507 (77.3)	1,170 (82.6)	969 (70.9)	1,213 (80.4)	1,117 (77.5)	1,038 (74.4)	
*Yes*	1,620 (22.7)	247 (17.4)	397 (29.1)	295 (19.6)	324 (22.5)	357 (25.6)	
In-hospital mortality							<0.001
*No*	5,502 (77.2)	1,168 (82.4)	968 (70.9)	1,212 (80.4)	1,117 (77.5)	1,037 (74.3)	
*Yes*	1,625 (22.8)	249 (17.6)	398 (29.1)	296 (19.6)	324 (22.5)	358 (25.7)	

BMI – body mass index, BUN – blood urea nitrogen, eGFR – estimated glomerular filtration rate, GCS – Glasgow Coma Scale, RRT – renal replacement therapy, SAPS II – Simplified Acute Physiology Score II, SOFA – Sequential Organ Failure Assessment, SpO_2_ – peripheral oxygen saturation, WBC – white blood cell count

*Values are presented as n (%) for categorical variables, mean (standard deviation) for normally distributed continuous variables, and median (interquartile range) for skewed continuous variables.

### Statistical analysis

Structured Query Language was used to extract data from the MIMIC-IV database. Variables with missing values accounting for more than 30% of the total sample size among vital signs and biochemical indicators were excluded. For serum sodium and UO included in trajectory modelling, we first summarised their missing data across the four-time windows (Table S1 in the **Online Supplementary Document**). Given the low missing rates of serum sodium and UO in all time windows, multiple imputation *via* the random forest algorithm implemented in the ‘mice’ package was adopted to handle missing serum sodium and UO values for the primary analysis, and the GBMTM model was constructed based on the imputed data sets. Missing values of covariates were processed following the identical multiple imputation protocol. Considering the potential presence of missing not at random (MNAR) in ICU data sets, three scenarios, including missing at random (MAR), moderate MNAR, and severe MNAR were further established for sensitivity analyses. The adjusted Rand index (ARI) and the magnitude of changes in primary effect sizes were calculated to evaluate the impact of distinct missing mechanism assumptions on the stability of trajectory clustering and risk estimation.

GBMTM was established using data from four 24-hour intervals (0–24 hours, 24–48 hours, 48–72 hours, 72–96 hours) within the first four days after ICU admission of sepsis patients. With time treated as the independent variable, classification schemes comprising 2 to 5 subtypes were explored under both linear and quadratic fitting models. Criteria for identifying the optimal model included a subtype proportion greater than 10%, an average posterior probability higher than 0.7, and the lowest possible Bayesian Information Criterion (BIC) and Akaike Information Criterion (AIC). Before analysis, z-score standardisation was performed on serum sodium and UO to eliminate dimensional differences and guarantee fairness and comparability of clustering results. To test the influence of the preset number of classes on classification outcomes, an alternative model with K = 4 classes was constructed. Models with K = 6 and K = 7 were further explored after relaxing the restriction that each class accounted for >10% of the total sample, to assess the stability of clustering structures.

The ‘tableone’ package was used to generate baseline characteristic tables. Normally or approximately normally distributed continuous variables were presented as mean ± standard deviation, with one-way analysis of variance adopted for intergroup comparisons. Skewed continuous variables were reported as median (interquartile range), and the Kruskal-Wallis test was used for between-group comparisons. Categorical variables were expressed as the number of cases (percentage), and the χ^2^ test or Fisher exact test was applied for intergroup comparisons. Kaplan-Meier survival curves were plotted to visualise the 30-day survival status of sepsis patients across distinct trajectory subtypes, and the log-rank test was used to compare survival disparities among subtypes. Cox proportional hazards models were adopted to evaluate associations between trajectory subtypes and clinical endpoints, including the primary endpoint (30-day mortality) and secondary endpoints (90-day mortality and in-hospital mortality). Covariates incorporated into models were selected based on previous prognostic studies of sepsis, clinical relevance, and potential confounders available in the baseline characteristic table. Three regression models were constructed sequentially: a crude unadjusted model, a minimally adjusted Model 1, and a fully adjusted Model 2. Model 1 was adjusted for age, sex and race; Model 2 additionally adjusted for BMI, timing of sepsis onset, heart rate, SpO_2_, lactate, anion gap, chloride, potassium, platelet count, pH, blood urea nitrogen, white blood cell count, baseline estimated glomerular filtration rate, fluid balance, calcium, invasive mechanical ventilation, β-blocker use, vasoactive agent administration, mannitol therapy, diuretic therapy, RRT, albumin infusion, Glasgow Coma Scale (GCS) score, Simplified Acute Physiology Score II (SAPS II) score, SOFA score, congestive heart failure, chronic pulmonary disease, acute liver failure, chronic kidney disease, diabetes mellitus and hypertension. Subgroup analyses and interaction term tests for key clinical characteristics were further conducted based on the fully adjusted model.

A series of supplementary analyses were performed to further evaluate the robustness of study findings and address methodological concerns regarding exposure definition, potential survivor bias, overfitting, covariate selection, and dynamic exposure modelling. Sensitivity analysis for exposure definition: the mean values of serum sodium and UO within each 24-hour time window replaced the minimum values adopted in the primary analysis. Group-based multi-trajectory modelling was refitted, and Cox regression analyses were repeated to assess how extraction strategies of exposure variables affected trajectory classification and prognostic associations. Sensitivity analysis for survivor bias: excluding patients with ICU length of stay <96 hours in the primary analysis might introduce survivor bias. An expanded cohort restricted to patients with ICU stay ≥48 hours (n = 12,506) was generated; GBMTM was refitted, and Cox regression was repeated to verify whether core conclusions remained stable after enrolling more patients with short ICU admission durations. Sensitivity analysis for covariate selection: Least Absolute Shrinkage and Selection Operator (LASSO) regression with 5-fold cross-validation was used to screen the 34 covariates included in the fully adjusted model. Core variables selected using the one-standard-error criterion were used to build a parsimonious Cox model, evaluating the sensitivity of primary effect sizes to covariate selection. Sensitivity analysis for time-varying exposure: time-varying Cox models were constructed, incorporating serum sodium and UO measurements from the four-time windows as time-dependent dynamic exposures. Cluster-robust standard errors were applied to correct correlations between repeated measurements from the same individual.

Additional analyses were carried out to assess the reproducibility, stability, and early risk-stratification value of the classification scheme. Early trajectory transition analysis: an early-stage GBMTM model was built solely using data from the first 48 hours to assign each patient an ‘early risk status’. A matrix transition comparison was performed between early risk status and the ‘final risk status’ defined by the complete 0–96 hours trajectories in the primary analysis, exploring associations between shifts in early risk status and 30-day survival outcomes. Reproducibility validation of clustering structure: a random forest algorithm was applied for 50 rounds of 70/30 random split-sample validation. Average classification accuracy and ARI were calculated to quantify the replicability of the five-subtype trajectory scheme across repeated sampling. Stability validation of prognostic associations: bootstrap resampling with 100 iterations was implemented for the primary Cox models, and 95% confidence intervals (CIs) of effect sizes were calculated to quantify random error in associations between trajectory subtypes and mortality risk.

All analyses were conducted using *R* software, version 4.4.1 (R Foundation for Statistical Computing, Vienna, Austria), with *P* < 0.05 denoting statistical significance.

## RESULTS

### Multivariable trajectory modelling analysis

During GBMTM model fitting, the log-likelihood increased as the number of subtypes increased. The five-subtype model yielded the lowest BIC = 123,812.84 and AIC = 123,476.13, representing the best balance between goodness-of-fit and complexity. Furthermore, the average posterior probabilities for subtypes 1–5 were all above the 70% threshold (86%, 89%, 83%, 85%, and 86%), indicating reliable classification (Table S2 in the **Online Supplementary Document**). Thus, the 5-subtype model was selected.

**Figure 2:**
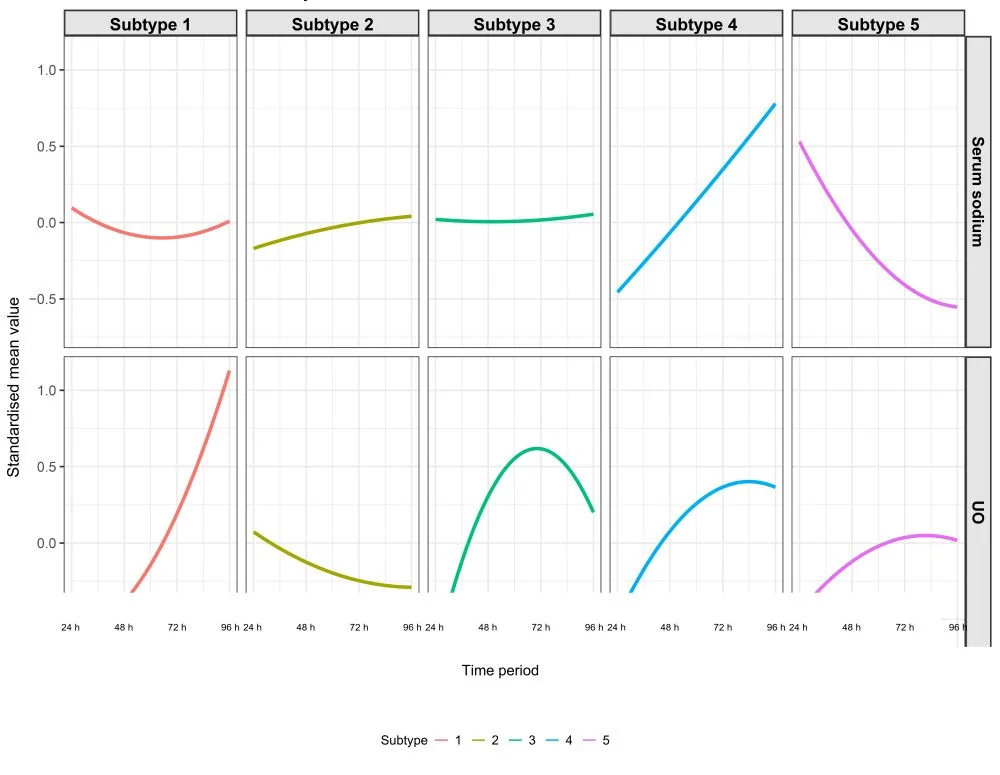
Dynamic trajectories of serum sodium and urine output within 96 hours of ICU admission in sepsis patients. ICU – intensive care unit, UO – urine output.

Five distinct dynamic trajectory patterns for serum sodium and UO within 96 hours after ICU admission were identified (**Figure 2**): subtype 1 (reference): mild initial serum sodium decrease followed by recovery, gradually increasing UO; subtype 2: mild serum sodium increase, gradually decreasing UO; subtype 3: relatively stable serum sodium, UO increasing then decreasing; subtype 4: gradually increasing serum sodium, UO increasing then stabilising; subtype 5: gradually decreasing serum sodium, UO increasing before levelling off. Combined plots showed lower UO at 96 hours in subtypes 2–5 compared with subtype 1 (Figure S1 in the **Online Supplementary Document**).

### Characteristics of the study population

The study included 7,127 sepsis patients with a slight male predominance (56.8% *vs.* 43.2%) and a mean age of 65.42 ± 16.61 years. Significant differences in 30-day mortality, 90-day mortality, and in-hospital mortality were observed across all trajectory subtypes (*P* < 0.001) (**Table 1**).

Analysis of actual serum sodium and UO distributions across the total population and subtypes revealed significant differences in serum sodium at 0–24 hours and 48–96 hours, and in UO across all time intervals (all *P* < 0.001) (Table S3 in the **Online Supplementary Document**).

### Survival analysis

**Figure 3:**
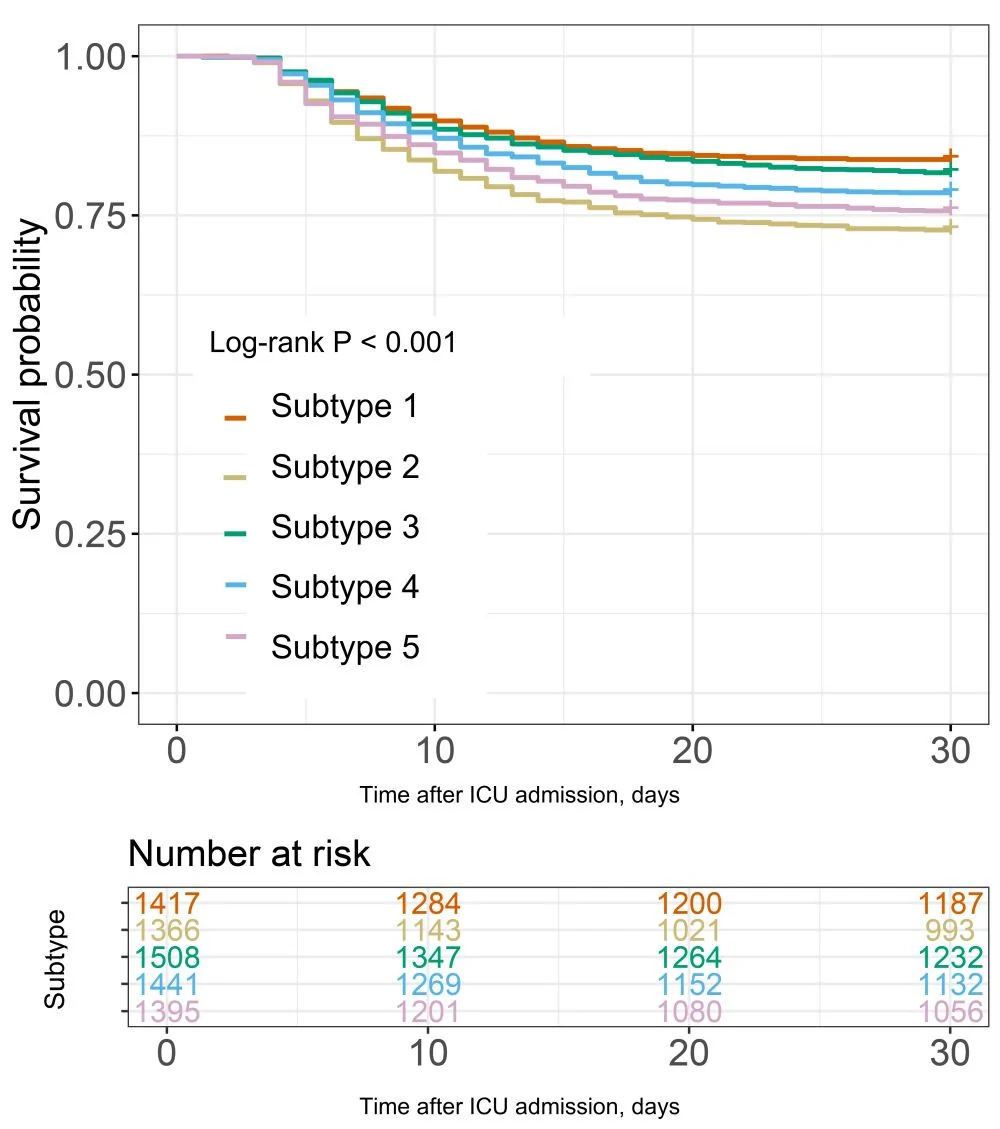
Kaplan-Meier survival curves.

Kaplan-Meier survival curves revealed an overall significant difference in 30-day survival rates among the five sepsis trajectory subtypes (log-rank *P* < 0.001), with subtype 2 exhibiting the lowest 30-day survival probability (**Figure 3**). Pairwise comparisons demonstrated that sepsis patients in subtype 2, subtype 4, and subtype 5 had markedly reduced 30-day survival relative to subtype 1 (all log-rank *P* < 0.001), whereas no statistically significant difference was detected between subtype 3 and Subtype 1 (log-rank *P* = 0.154) (Table S4 in the **Online Supplementary Document**). In addition, the differences between subtype 2 and subtype 5 (log-rank *P* = 0.070), as well as between subtype 4 and subtype 5 (log-rank *P* = 0.057), failed to reach statistical significance (Table S4 in the **Online Supplementary Document**). These findings suggested partial similarity or overlap in mortality risk across certain trajectory subtypes.

### Association between sepsis subtypes and prognosis

In the fully adjusted model, relative to subtype 1, subtypes 2 exhibited 85.0% higher 30-day mortality (HR = 1.850; 95% CI = 1.563–2.189, *P* < 0.001), while subtypes 3, 4, and 5 exhibited 22.2% (HR = 1.222; 95% CI = 1.025–1.458, *P* = 0.026), 30.9% (HR = 1.309; 95% CI = 1.099–1.558, *P* = 0.003), and 53.0% (HR = 1.530; 95% CI = 1.290–1.815, *P* < 0.001) higher 30-day mortality, respectively (**Table 2**).

**Table 2:** Association between sepsis subtypes and 30-day mortality

Characteristic	HR (95% CI), *P*-value		
	Crude model*	Model 1*	Model 2*
30-day mortality			
Subtype 1	Ref.	Ref.	Ref.
Subtype 2	1.804 (1.531–2.126), <0.001	1.823 (1.546–2.148), <0.001	1.850 (1.563–2.189), <0.001
Subtype 3	1.135 (0.953–1.352), 0.156	1.120 (0.940–1.334), 0.205	1.222 (1.025–1.458), 0.026
Subtype 4	1.355 (1.142–1.607), <0.001	1.353 (1.140–1.605), <0.001	1.309 (1.099–1.558), 0.003
Subtype 5	1.574 (1.331–1.861), <0.001	1.538 (1.301–1.818), <0.001	1.530 (1.290–1.815), <0.001


BMI – body mass index, GCS – Glasgow coma scale, pH – potential of hydrogen, SAPS II – simplified acute physiology score II, SOFA – sequential organ failure assessment, SpO2 – peripheral oxygen saturation

Crude model: unadjusted. Model 1: adjusted for age, race, and sex. Model 2: fully adjusted for age, race, sex, BMI, sepsis onset timing (pre-ICU/post-ICU), heart rate, SpO2, lactate, anion gap, chloride, potassium, platelet count, pH, blood urea nitrogen, white blood cell count, baseline estimation of glomerular filtration rate, fluid balance, calcium, invasive ventilation, β-blockers, vasoactive drugs, mannitol treatment, diuretic therapy, renal replacement therapy, albumin infusion, GCS, SAPS II, SOFA score, congestive heart failure, chronic pulmonary disease, acute liver failure, renal disease, diabetes, and hypertension.

Analysis of secondary outcomes yielded consistent findings: in the fully adjusted model, subtypes 2 (90-day mortality: HR = 1.843, *P* < 0.001; in-hospital mortality: HR = 1.834, *P* < 0.001), 3 (90-day mortality: HR = 1.218, *P* = 0.024; in-hospital mortality: HR = 1.211, *P* = 0.027), 4 (90-day mortality: HR = 1.296, *P* = 0.003; in-hospital mortality: HR = 1.285, *P* = 0.004), and 5 (90-day mortality: HR = 1.490, *P* < 0.001; in-hospital mortality: HR = 1.480, *P* < 0.001) were associated with significantly increased 90-day and in-hospital mortality compared with Subtype 1 (Table S5 in the **Online Supplementary Document**).

### Subgroup analysis

Subgroup analyses indicated significant interaction effects in BMI (*P* for interaction = 0.031), RRT (*P* for interaction = 0.007), albumin infusion (*P* for interaction = 0.009), and vasoactive drug (*P* for interaction = 0.043) subgroups (**Table 3**).

**Table 3:** Subgroup analysis of the association between sepsis subtypes and 30-day mortality

Characteristics	HR (95% CI), *P*-value					*P* for interaction
	**Subtype 1**	**Subtype 2**	**Subtype 3**	**Subtype 4**	**Subtype 5**	
Sex						0.132
*Female*	Ref.	2.390 (1.850–3.087), <0.001	1.420 (1.082–1.864), 0.011	1.298 (0.981–1.718), 0.067	1.749 (1.345–2.273), <0.001	
*Male*	Ref.	1.509 (1.203–1.892), <0.001	1.086 (0.860–1.373), 0.488	1.282 (1.024–1.605), 0.031	1.382 (1.101–1.734), 0.005	
BMI						0.031
*≤25 kg/m^2^*	Ref.	2.123 (1.592–2.831), <0.001	1.518 (1.125–2.048), 0.006	1.399 (1.026–1.909), 0.034	1.508 (1.115–2.039), 0.008	
*25-30 kg/m^2^*	Ref.	1.516 (1.123–2.048), 0.007	1.030 (0.757–1.401), 0.853	1.024 (0.749–1.400), 0.881	1.235 (0.911–1.674), 0.174	
*>30 kg/m^2^*	Ref.	1.797 (1.332–2.426), <0.001	1.118 (0.812–1.539), 0.495	1.536 (1.140–2.068), 0.005	1.880 (1.410–2.506), <0.001	
RRT						0.007
*No*	Ref.	2.060 (1.712–2.480), <0.001	1.232 (1.018–1.491), 0.032	1.266 (1.048–1.529), 0.014	1.471 (1.216–1.781), <0.001	
*Yes*	Ref.	1.240 (0.814–1.887), 0.316	1.246 (0.762–2.039), 0.381	1.439 (0.887–2.336), 0.141	1.661 (1.101–2.507), 0.016	
Diuretic therapy						0.056
*No*	Ref.	1.754 (1.369–2.248), <0.001	1.259 (0.970–1.633), 0.083	1.406 (1.086–1.820), 0.010	1.656 (1.294–2.119), <0.001	
*Yes*	Ref.	1.880 (1.490–2.372), <0.001	1.153 (0.905–1.468), 0.249	1.170 (0.920–1.488), 0.200	1.344 (1.055–1.711), 0.017	
Albumin infusion						0.009
*No*	Ref.	1.725 (1.423–2.090), <0.001	1.135 (0.926–1.392), 0.224	1.089 (0.890–1.334), 0.407	1.420 (1.164–1.731), <0.001	
*Yes*	Ref.	2.186 (1.530–3.125), <0.001	1.639 (1.138–2.361), 0.008	1.971 (1.371–2.833), <0.001	2.058 (1.454–2.915), <0.001	
Vasoactive drugs						0.043
*No*	Ref.	1.936 (1.499–2.501), <0.001	1.415 (1.081–1.851), 0.011	1.559 (1.203–2.021), <0.001	1.375 (1.037–1.823), 0.027	
*Yes*	Ref.	1.824 (1.454–2.289), <0.001	1.082 (0.854–1.371), 0.514	1.075 (0.845–1.368), 0.556	1.651 (1.330–2.051), <0.001	

BMI – body mass index, CI – confidence interval, HR – hazard ratio, RRT – renal replacement therapy

Compared to subtype 1, among patients with BMI≤25 kg/m^2^, subtypes 3 (HR = 1.518, *P* = 0.006), 4 (HR = 1.399, *P* = 0.034), and 5 (HR = 1.508, *P* = 0.008) had elevated 30-day mortality; among patients with BMI>30 kg/m^2^, subtypes 4 (HR = 1.536, *P* = 0.005) and 5 (HR = 1.880, *P* < 0.001) had increased mortality. subtype 2 was associated with increased mortality across all BMI categories (*P* < 0.05) (**Table 3**).

Furthermore, relative to subtype 1, subtype 5 exhibited significantly higher mortality independent of RRT status (*P* < 0.05). Similarly, subtypes 2 and 5 were linked to increased mortality regardless of albumin infusion or vasoactive drug administration (*P* < 0.05) (**Table 3**).

### Sensitivity analyses and model validation

Multiple sensitivity analyses indicated that the primary findings were generally robust. Under distinct missing-data mechanism assumptions, the ARI for the moderate MNAR and severe MNAR scenarios relative to MAR were 0.950 and 0.925, respectively, with a proportion of reclassified participants of 2.06% and 3.09%. The maximum magnitude of change in key Cox regression effect sizes was only 2.74%, suggesting that assumptions regarding missing-data mechanisms exerted minimal influence on the core results (Tables S1, S6–7 in the **Online Supplementary Document**). When mean values within each time window replaced the minimum values used in the primary analysis, the overall trajectory patterns remained consistent with the main model. In the fully adjusted Cox model, subtype 2, subtype 4 and subtype 5 still conferred elevated mortality risks *vs.* subtype 1, while subtype 3 exhibited a borderline significant trend (Figure S2 and Table S8 in the **Online Supplementary Document**). Analyses of alternative class numbers demonstrated that the K = 4 model mainly aggregated similar trajectories identified in the K = 5 model rather than generating entirely distinct trajectory structures (Figures S3–4 and Table S9 in the **Online Supplementary Document**). The K = 6 model did not yield a lower BIC compared with the K = 5 model. Although the K = 7 model presented a lower BIC, it contained small subgroups and the minimum average posterior probability declined to 0.69, indicating insufficient certainty for this classification scheme (Figure S5 and Table S10 in the **Online Supplementary Document**).

Additional robustness analyses based on the expanded cohort restricted to patients with an ICU length of stay ≥48 hours reproduced analogous trajectory structures. Subtype 2, subtype 3, and subtype 4 remained significantly associated with higher 30-day mortality relative to subtype 1 (HR range = 1.660–1.839, *P* < 0.001), whereas the association for subtype 5 lost statistical significance (Figure S6 and Table S11 in the **Online Supplementary Document**). The parsimonious Cox model after LASSO variable selection showed consistent directions of associations between each trajectory subtype and 30-day mortality risk as observed in the fully adjusted model (Figure S7 and Table S12 in the **Online Supplementary Document**). In the time-varying Cox model, hazard ratios for trajectory subgroups were slightly attenuated compared with those from the original Cox model; nevertheless, subtype 2 and subtype 5 still carried significantly increased mortality risk *vs.* subtype 1. Dynamic exposure analyses revealed that higher UO was correlated with reduced mortality risk, while both hyponatraemia and hypernatraemia were associated with elevated mortality risk (Tables S13–14 in the **Online Supplementary Document**).

Internal validation and early trajectory transition analysis further supported the stability of trajectory stratification and its value for dynamic risk identification. The sample sizes of the five trajectory subgroups were relatively balanced, and their 30-day mortality rates presented a graded distribution, whereas SOFA scores were not perfectly synchronised with mortality risks (Table S15 in the **Online Supplementary Document**). In random split-sample validation, the average prediction accuracy of the five-subtype trajectory labels reached 0.759, with an ARI = 0.493. Bootstrap resampling of Cox regression models demonstrated consistent hazard ratio directions for all high-risk subtypes relative to subtype 1 (Table S16 in the **Online Supplementary Document**). Early 48-hour trajectory transition analysis revealed that the 30-day mortality rate of the ‘improved’ subgroup was 16.96%, which was comparable to that of the persistently low/moderate-risk subgroup (17.36%). Both the worsening subgroup and persistently high-risk subgroup exhibited elevated 30-day mortality risks compared with the persistently low/moderate-risk subgroup (Table S17 in the **Online Supplementary Document**). Among patients classified as early high-risk, those with sustained high trajectory risk carried a higher mortality hazard relative to patients whose trajectories improved (HR = 1.73; 95% CI = 1.36–2.21, *P* < 0.001) (Table S18 in the **Online Supplementary Document**). These findings indicate that early trajectory shifts possess potential value for dynamic prognostic risk discrimination; however, causal inference that trajectory modification or clinical interventions can reduce mortality risk cannot be drawn directly.

## DISCUSSION

This study, based on the MIMIC-IV database applied GBMTM to identify five distinct subtypes of combined dynamic trajectories of serum sodium and UO within the first 96 hours after ICU admission among patients with sepsis. Primary analyses demonstrated that subtypes 2, 3, 4 and 5 were all associated with elevated risks of 30-day, 90-day and in-hospital mortality compared with subtype 1. Of these subgroups, subtype 2 exhibited the most consistent excess mortality risk, while subtype 5 also conferred substantially higher mortality across most analytical frameworks. Multiple sensitivity analyses confirmed overall consistency of the core results under alternative assumptions regarding missing data mechanisms, exposure variable definitions, trajectory class specifications, ICU length of stay thresholds, covariate selection strategies, and time-varying exposure modelling. Nevertheless, the magnitude of hazard ratios for several subtypes was moderately attenuated in the expanded ICU cohort and time-varying Cox models. Collectively, these observations support moderate robustness of the association between trajectory subtypes and mortality risk, though all interpretations must be tempered by the inherent limitations of an observational study design. Significant effect modification was detected across subgroups stratified by BMI, RRT, albumin infusion, and vasoactive agent administration. This interaction evidence indicated that the prognostic implication of serum sodium-UO trajectories may differ according to patients’ distinct clinical backgrounds.

The present study demonstrated that sepsis patients in subtype 1 had relatively favourable prognosis, whereas subtype 2 exhibited the highest 30-day mortality. Subtype 2 was characterised by a sustained decline in UO throughout the 0–96 hours period following ICU admission, suggesting that persistent oliguria may serve as a vital prognostic marker reflecting inadequate renal perfusion, impaired renal function, or overall disease severity [12,13]. A previous study has indicated that sepsis-induced insufficient renal perfusion, inflammation and oxidative stress may collectively contribute to decreased UO and the development of AKI [14]. Sepsis patients complicated with AKI also carry a higher mortality risk [15]. Accordingly, the trajectory of persistently declining UO represented by subtype 2 may reflect a high-risk state characterised by poor recovery of renal perfusion and organ function in the early phase of sepsis. It should be emphasised that this study is an observational trajectory analysis; decreased UO should be interpreted merely as a biomarker of high-risk physiological status, and we cannot directly infer that simply elevating UO or altering UO trajectories would improve survival outcomes.

Although UO in sepsis patients of subtype 4 and subtype 5 both rose and then plateaued, their serum sodium levels deviated from subtype 1 in opposite directions: serum sodium gradually increased in subtype 4 while it progressively decreased in subtype 5. Both hyponatraemia and hypernatraemia are associated with elevated mortality risk among patients with sepsis [16]. Abnormal serum sodium levels may correlate with adverse outcomes *via* multiple pathways, including disrupted osmotic balance, altered neurological function, fluctuating volume status, and impaired organ perfusion. Furthermore, disturbances in sodium homeostasis may interact with inflammatory and immunomodulatory processes [17–19]. Nevertheless, the present study did not directly measure these underlying mechanisms. Accordingly, the association between aberrant serum sodium trajectories and elevated mortality risk should be primarily interpreted as a prognostic biomarker signal. The relevant pathophysiological mechanisms remain explanatory hypotheses derived from prior literature and require further prospective and mechanistic investigations for validation.

Subgroup analyses revealed that BMI, RRT, albumin infusion, and vasoactive agents may modify the statistical association between trajectory subtypes and 30-day mortality risk. These findings indicate that the prognostic value of combined serum sodium-UO trajectories varies across patients with distinct baseline metabolic profiles, renal supportive interventions, and circulatory support backgrounds. Previous systematic reviews and meta-analyses have also demonstrated that the relationship between BMI and prognosis in patients with sepsis or septic shock is nonlinear and heterogeneous [20,21]. For instance, patients receiving RRT, vasoactive drugs, or albumin infusion often present complex volume status, renal impairment, and intensive circulatory support requirements. Vasoactive agents and albumin are tightly linked to fluid resuscitation and haemodynamic stabilisation in sepsis and septic shock, whereas RRT generally signifies advanced renal dysfunction or a greater demand for organ support [22]. These clinical conditions may confound the relationship between serum sodium-UO trajectories and mortality risk. Nevertheless, subgroup analyses in this study are exploratory by nature. Therapeutic variables, including albumin infusion, vasoactive medications, diuretics, and RRT, lie along complex causal pathways connecting disease severity, trajectory alterations, and mortality endpoints [23,24]. Accordingly, specific trajectory patterns should not be interpreted as a marker of poor responsiveness to standard care or a definitive therapeutic target. Instead, these trajectories are best regarded as high-risk prognostic phenotypes that warrant intensified clinical monitoring and further mechanistic research for validation.

Notably, pairwise survival comparisons revealed non-significant log-rank differences between subtype 1 and subtype 3, subtype 2 and subtype 5, as well as subtype 4 and subtype 5, indicating that certain trajectory subtypes may occupy adjacent or partially overlapping positions on the mortality risk spectrum. This finding suggests that trajectory stratification does not guarantee distinct, clinically independent disparities between every pair of subtypes. Instead, combined serum sodium-UO trajectories serve optimally as a prognostic risk-stratification framework to categorise patients into relatively low-risk, persistently high-risk, and intermediate overlapping risk groups, rather than being overinterpreted as completely discrete patient subgroups requiring disparate targeted therapies.

Early 48-hour trajectory transition analyses further illustrated that early high-risk status does not represent a fixed, irreversible prognostic category. The 30-day mortality rate of patients with improving trajectories was comparable to that of the persistently low/moderate-risk subgroup, while individuals with sustained high-risk trajectories faced substantially elevated mortality hazards. These observations support the utility of combined serum sodium-UO trajectories for dynamic early risk detection, which may facilitate timely identification of patients requiring close ongoing clinical surveillance. Nonetheless, this analysis relies exclusively on observational data and shifts in early risk trajectories can be jointly modulated by baseline disease severity, fluid management, renal function fluctuations, therapeutic regimens, and unmeasured confounders. Accordingly, we cannot establish causal links between clinical interventions and trajectory improvement, nor can we confirm that deliberate modification of these trajectories reduces mortality risk. Prospective cohort investigations are warranted to validate the reproducibility of early trajectory shifts and quantify their incremental predictive value for serial risk monitoring.

This study has several inherent limitations. First, all data were extracted from ICU patients at a single medical centre in the USA, with no independent external cohort validation. Caution is therefore advised when generalising these conclusions to other healthcare facilities or broader patient populations. Second, this is a retrospective observational study. Although multivariate Cox regression, LASSO-reduced models, time-varying Cox frameworks, and multiple sensitivity analyses were implemented to assess result robustness, residual unmeasured confounding, selection bias, and the mediating role of therapeutic variables along causal pathways cannot be fully eliminated. Causal inferences that trajectory modification or specific interventions directly lower mortality risk remain unsupported. Third, the primary analysis restricted enrolment to patients with an ICU length of stay ≥96 hours, which may exclude individuals who died early or were transferred out of the ICU prematurely and thereby introduce survivor bias. While sensitivity analyses using an expanded cohort of patients with an ICU length of stay ≥48 hours largely reproduced the core associations, this potential bias requires more rigorous mitigation in future research. Fourth, non-random missingness of serum sodium and UO measurements cannot be ruled out. Despite low overall missing rates and consistent findings across MAR/MNAR sensitivity scenarios, multiple imputation models rely on observed covariates and pre-specified missing-data assumptions. Finally, sepsis constitutes a highly heterogeneous syndrome driven by diverse infectious sources and pathogenic microorganisms [25]. This study did not conduct stratified trajectory modelling by infection site, pathogen category, or detailed therapeutic strategies, which may obscure divergent associations between longitudinal biomarker patterns and prognosis across distinct aetiological backgrounds.

## CONCLUSIONS

Based on the MIMIC-IV database, this study identified five distinct subtypes of combined longitudinal serum sodium and UO trajectories among sepsis patients. Compared with Subtype 1, the other four subtypes were generally associated with elevated risks of 30-day, 90-day and in-hospital mortality. However, pairwise survival comparisons indicated non-significant mortality differences between several pairs of subtypes. Subtype 2 (characterised by progressively declining UO) exhibited the most consistent excess mortality risk, and subtype 5 (marked by gradually decreasing serum sodium) also carried a high mortality risk across most analytical models, indicating that these two trajectory phenotypes are valuable for early prognostic risk stratification. Given the observational nature of this study, the above findings should primarily be used for risk stratification and hypothesis generation for future research, rather than being directly interpreted as therapeutic targets or evidence supporting treatment efficacy.

## Additional material


Online Supplementary Document

## Data Availability

**Data availability**: All the data within this manuscript could be gotten from corresponding author at reasonable request.
