# Supplementary material for: Dynamic trajectories of serum sodium and urine output and their association with prognosis in sepsis: a group-based multi-trajectory modelling analysis using MIMIC-IV
Source: J Glob Health. 2026 Sep 4;16:04308. doi: 10.7189/jogh.16.04308 (PMC13543246; doi:10.7189/jogh.16.04308)
Supplement: Supplementary file 1 [file jogh-16-04308-s001.pdf]

Supplement to: Zhuo X, Xu X, Chen H. Dynamic trajectories of serum sodium and urine output and their association with prognosis in sepsis: a group-based multi-trajectory modelling analysis using MIMIC-IV. J Glob Health. 2026;16:04308.

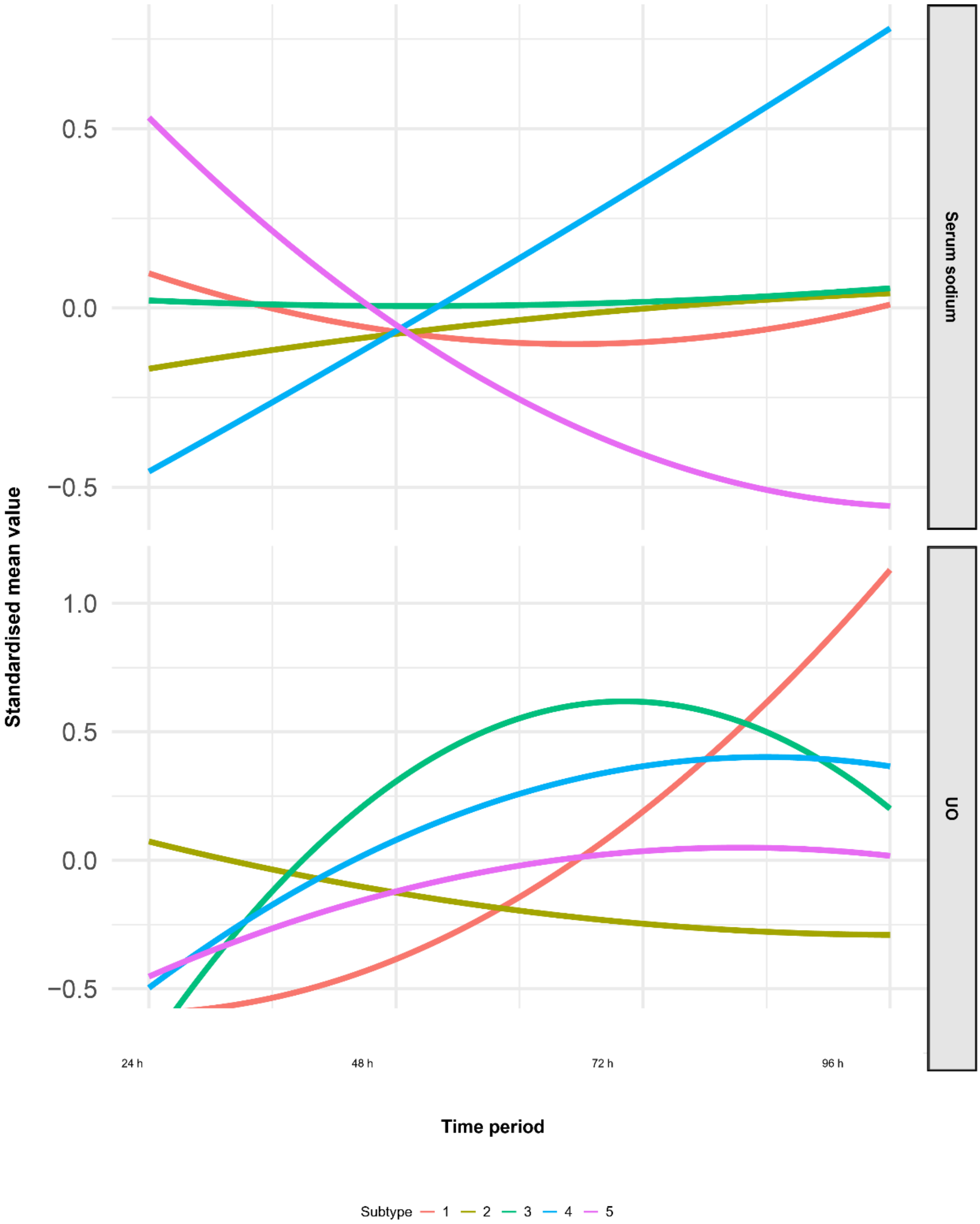

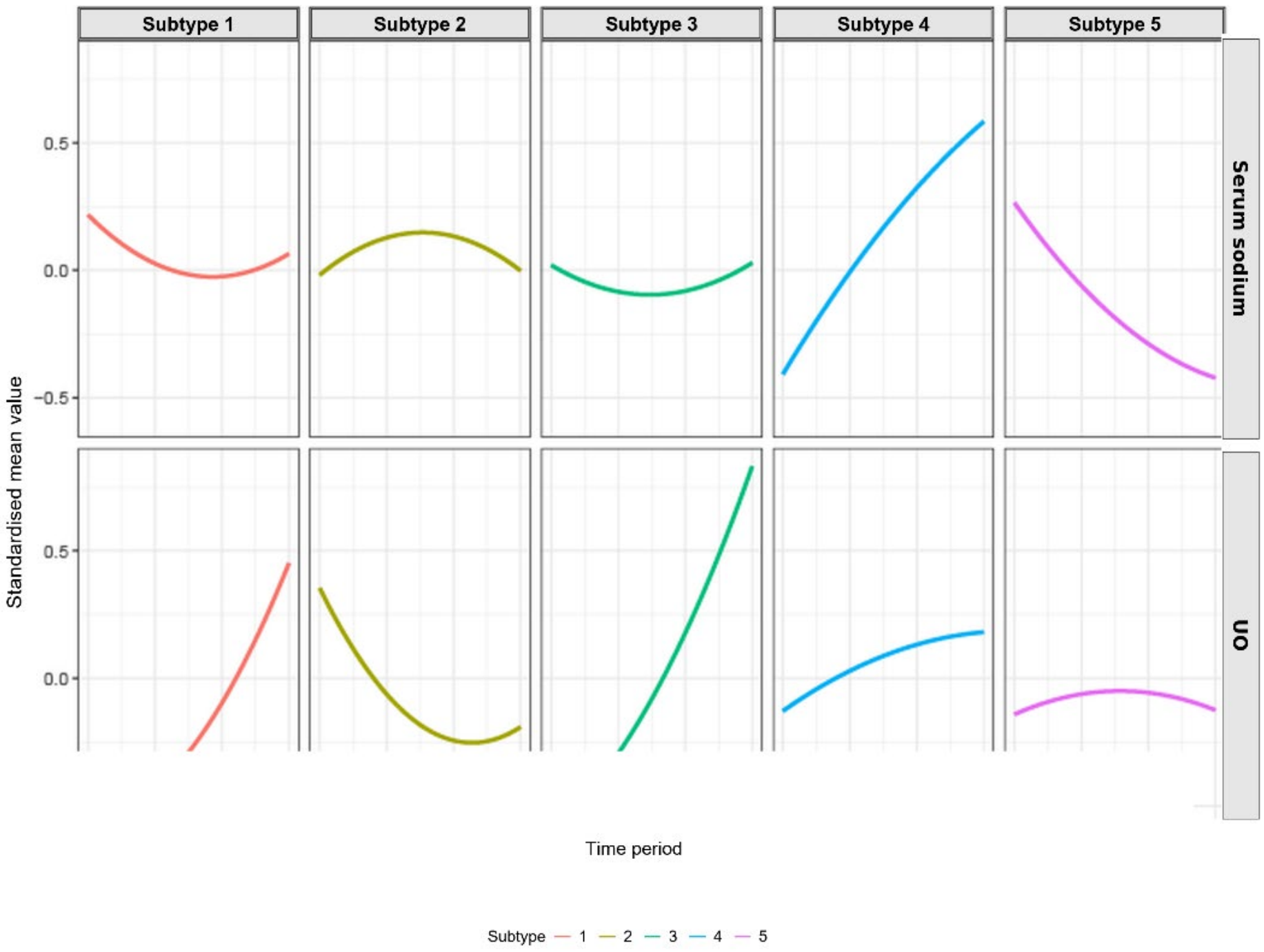

Subtype 1
Subtype 2
Subtype 3
Subtype 4
Subtype 5
Serum sodium
UO
Standardised mean value
0.5
0.0
-0.5
Time period
Subtype 1 2 3 4 5

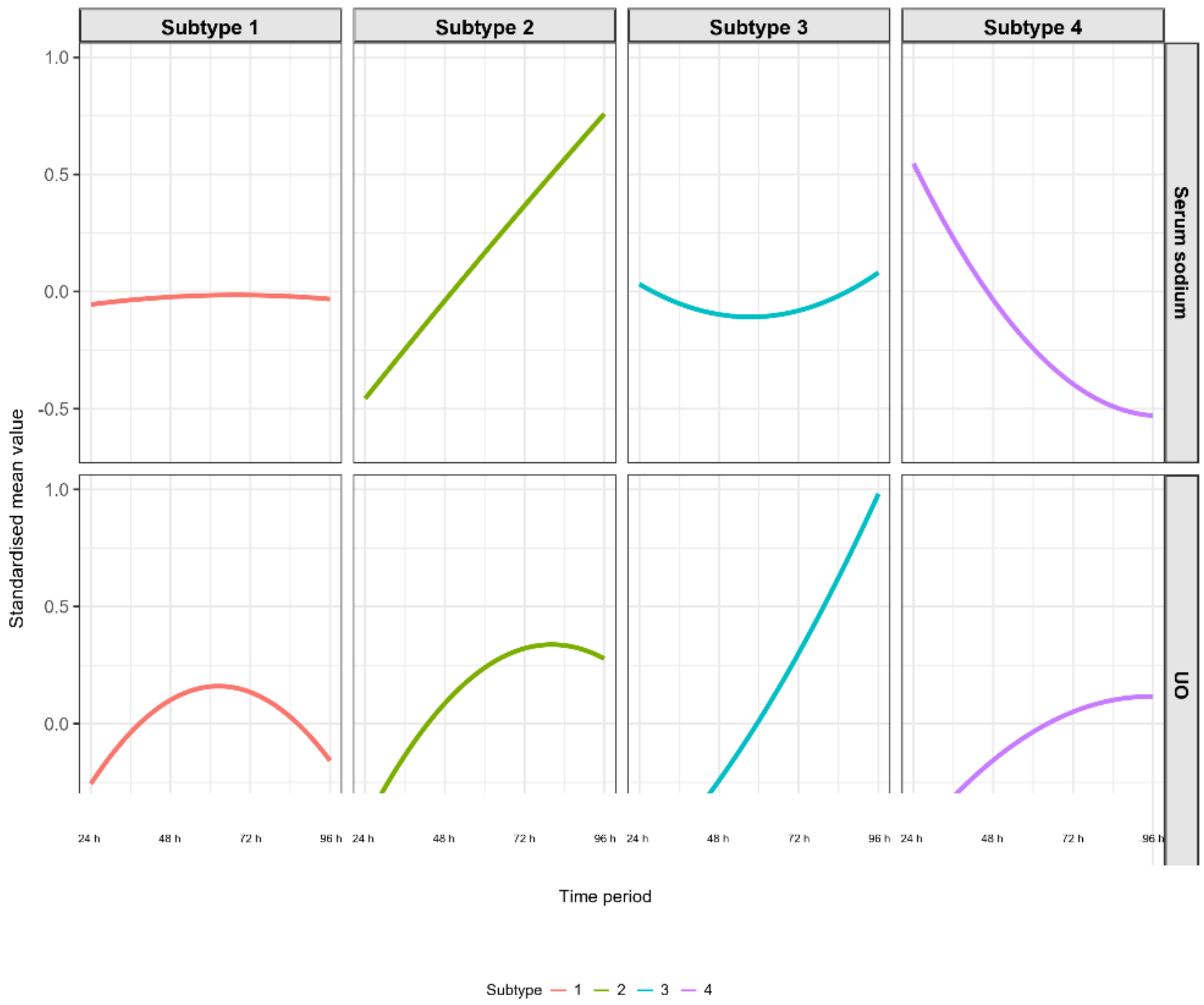

Subtype 1
Subtype 2
Subtype 3
Subtype 4
Serum sodium
UO
Standardised mean value
1.0
0.5
0.0
-0.5
24 h
48 h
72 h
96 h
Time period
Subtype 1 2 3 4

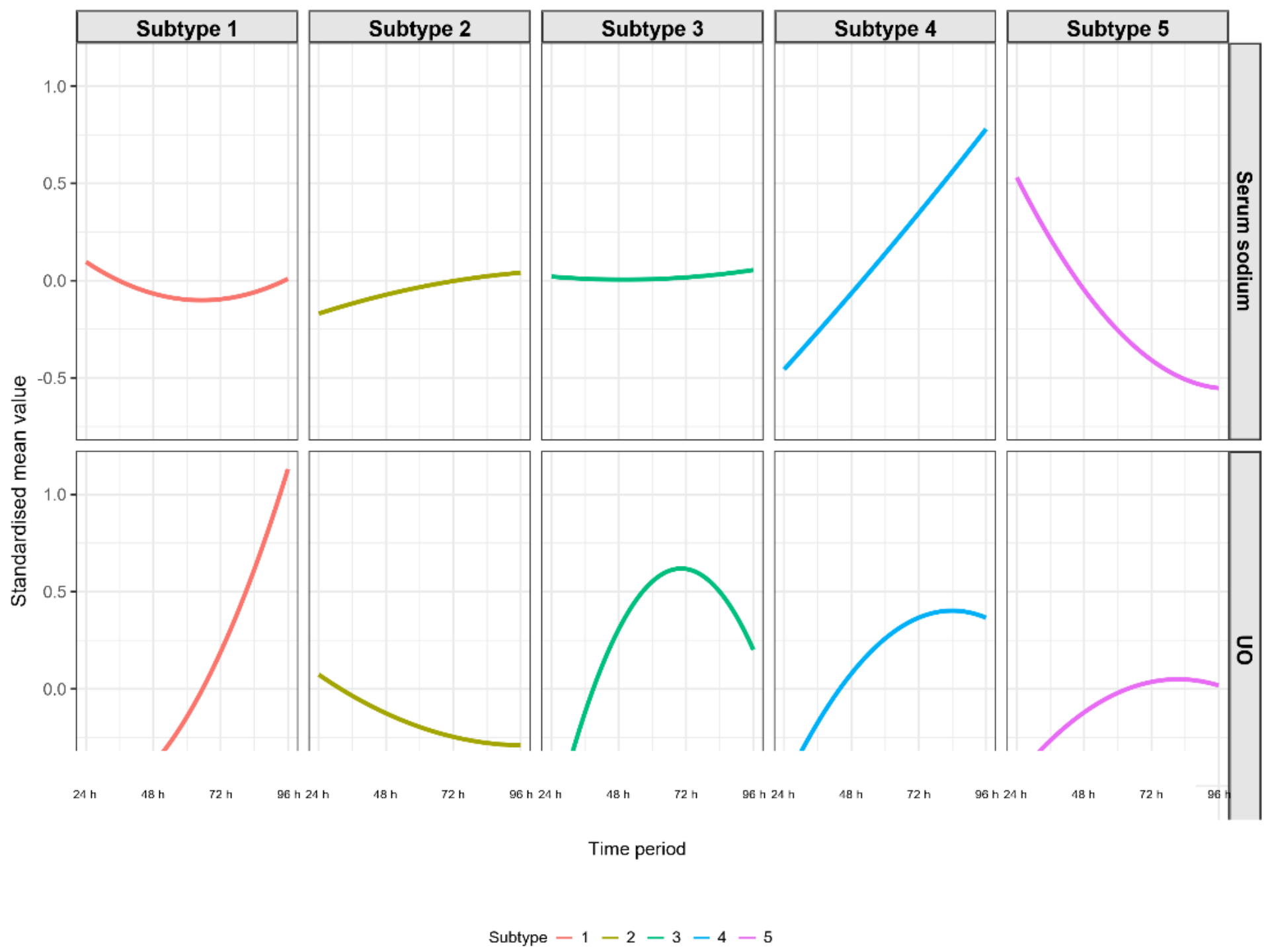

Subtype 1
Subtype 2
Subtype 3
Subtype 4
Subtype 5
Serum sodium
UO
Standardised mean value
Time period
Subtype 1 2 3 4 5

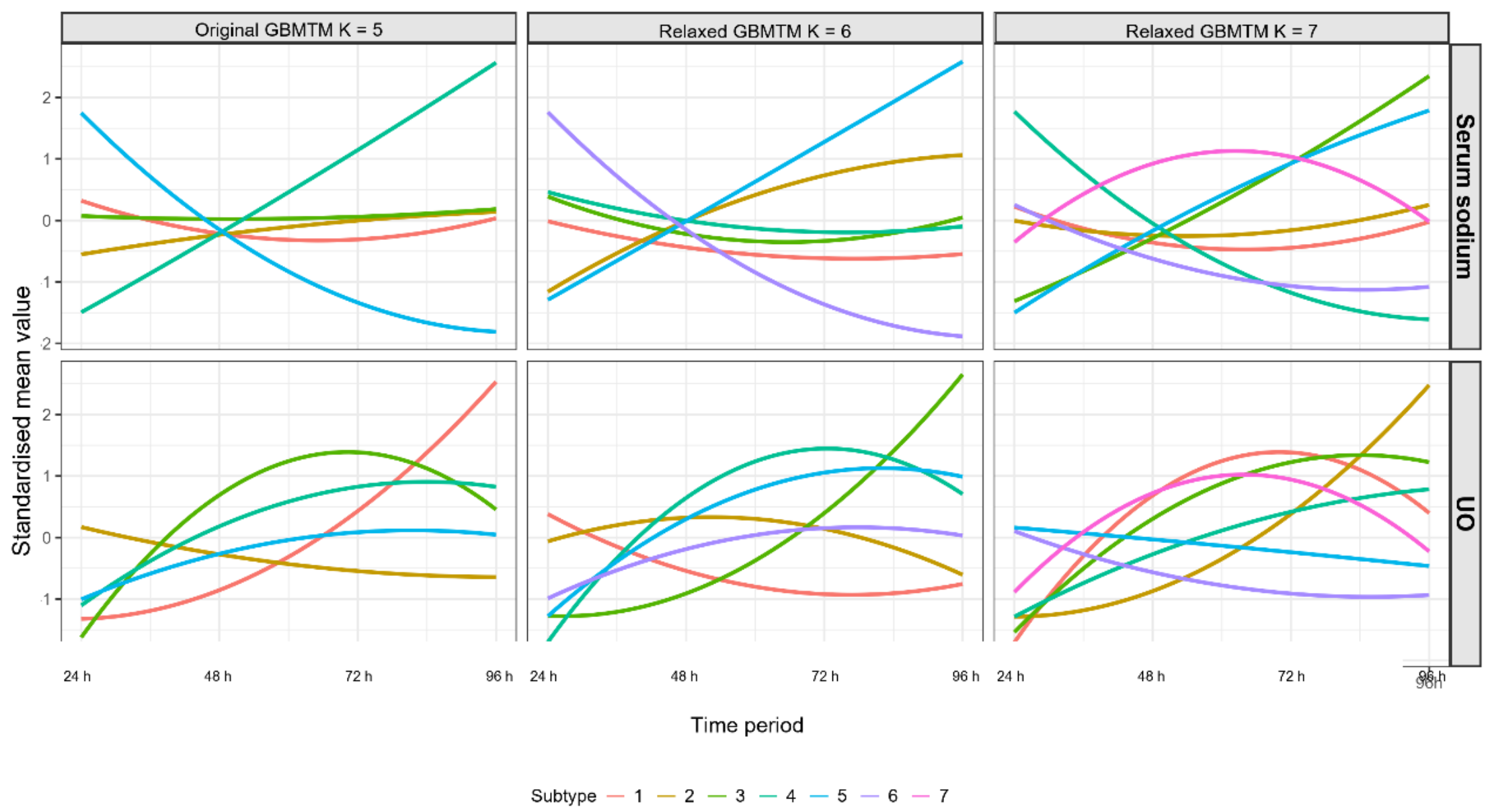

Original GBMTM K = 5
Relaxed GBMTM K = 6
Relaxed GBMTM K = 7
Serum sodium
UO
Standardised mean value
Time period
Subtype 1 2 3 4 5 6 7

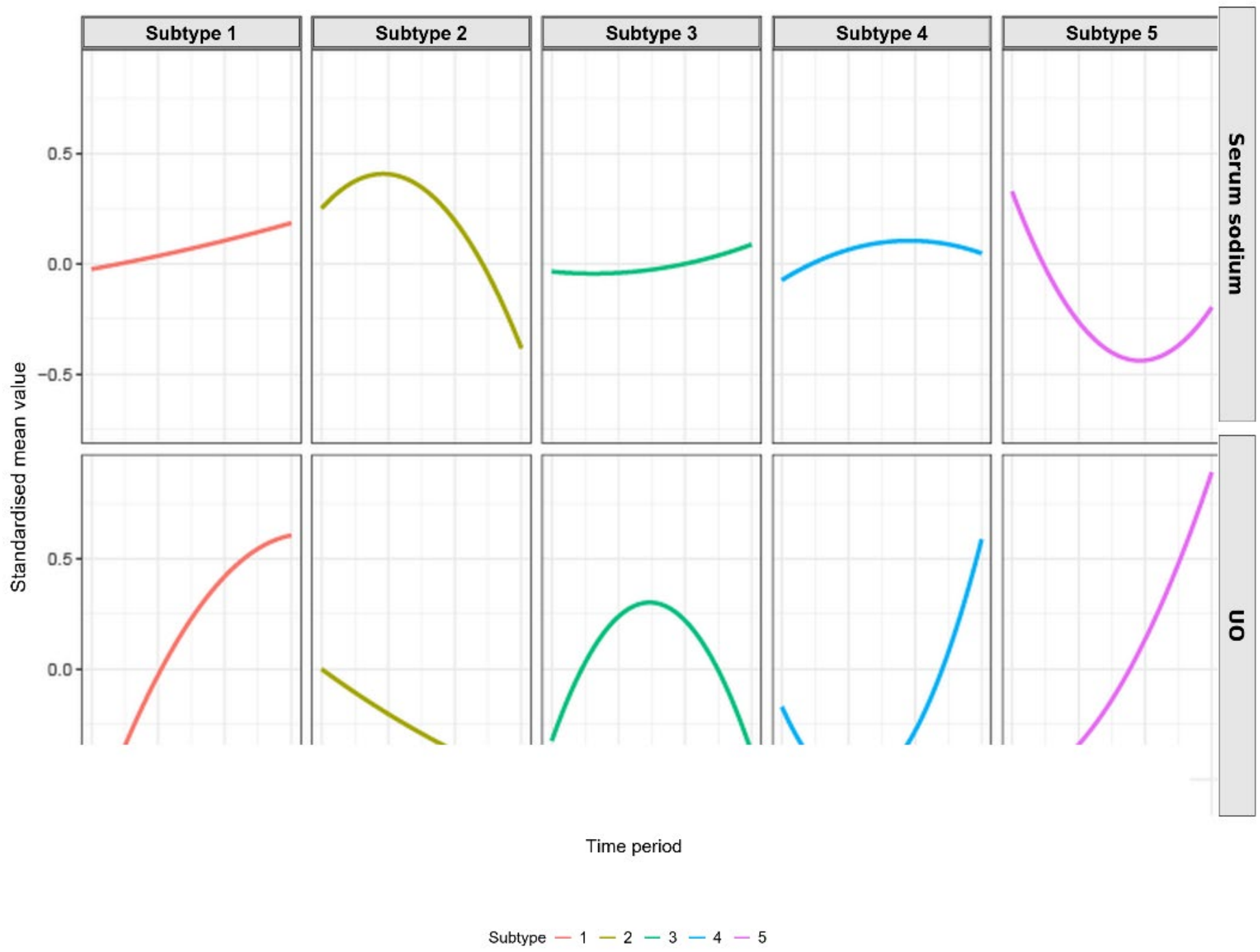

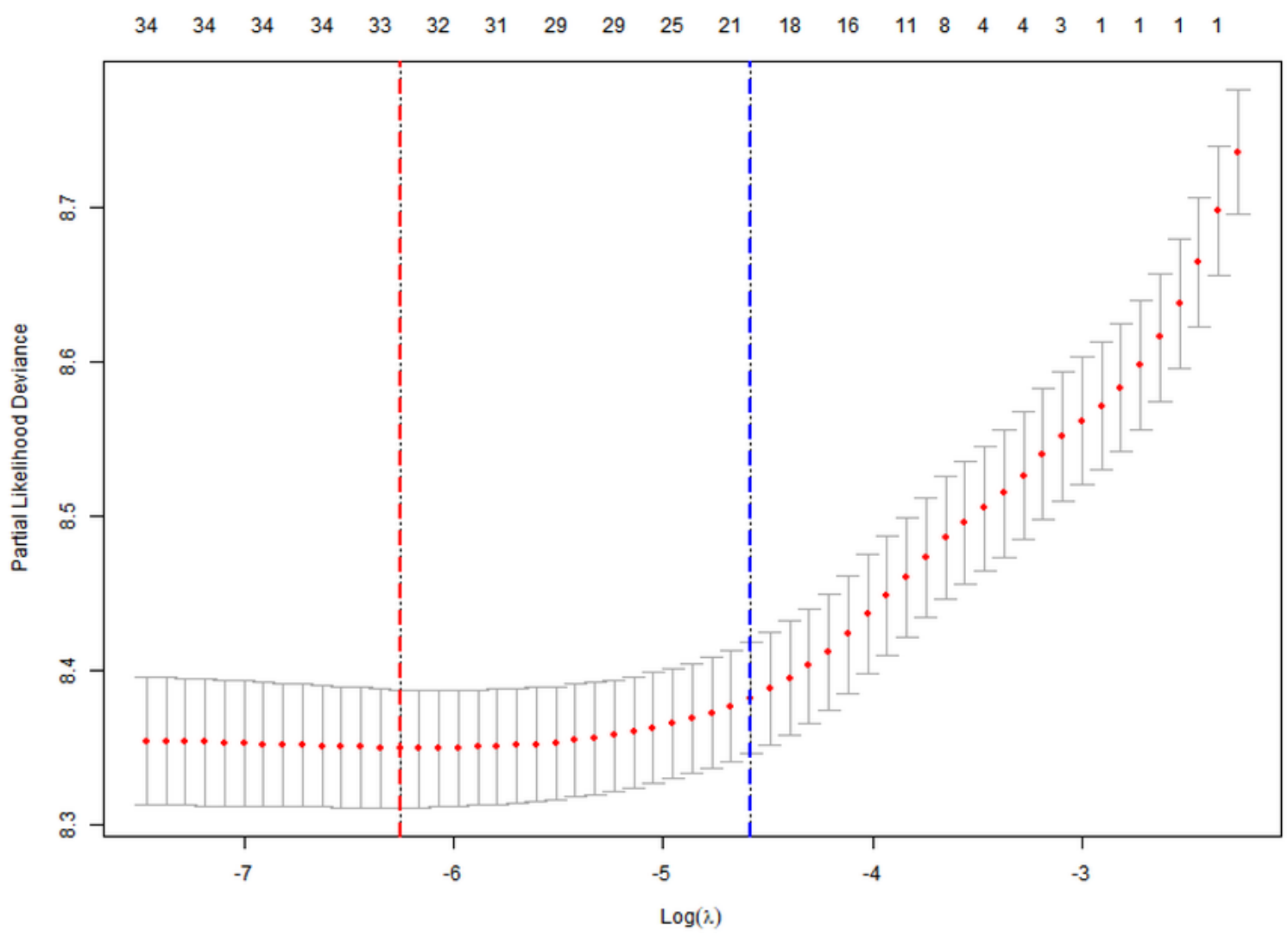

Table S1. Missingness of serum sodium and urine output across the four time windows.

Variable	Time window	Number missing (n)	Missing rate (%)
**Serum sodium**	0–24 h	32	0.45
Serum sodium	24–48 h	85	1.19

Serum sodium	48–72 h	88	1.23
Serum sodium	72–96 h	178	2.50
Urine output	0–24 h	140	1.96
Urine output	24–48 h	185	2.60
Urine output	48–72 h	216	3.03
Urine output	72–96 h	241	3.38

Percentages were calculated using the total study population as the denominator (N = 7,127).
h – hours.

Table S2. Group-based multi-trajectory modelling analysis.

Number of classes	Log-likelihood	AIC	BIC	Class proportions					Average posterior probability				
				1	2	3	4	5	1	2	3	4	5
Linear													
2	-66846.61	133723.23	133826.30	0.42	0.58				0.95	0.96			
3	-65023.82	130093.65	130251.70	0.31	0.39	0.30			0.90	0.92	0.85		
4	-63760.95	127583.91	127796.93	0.24	0.24	0.31	0.22		0.86	0.83	0.88	0.85	
5	-63407.69	126891.38	127152.50	0.18	0.25	0.23	0.17	0.16	0.83	0.84	0.87	0.83	0.80
Quadratic													
2	-66327.66	132693.32	132823.89	0.5	0.44				0.96	0.95			
3	-64469.86	128997.73	129197.01	0.37	0.31	0.32			0.92	0.87	0.91		
4	-62916.22	125910.43	126178.43	0.31	0.25	0.21	0.22		0.90	0.85	0.86	0.86	
5	-61689.07	123476.13	123812.84	0.20	0.19	0.21	0.20	0.20	0.86	0.89	0.83	0.85	0.86

AIC, Akaike Information Criterion; BIC, Bayesian Information Criterion.

Table S3. Distribution of serum sodium and urine output across time intervals by sepsis subtype after admission to the ICU.

Characteristics	Total (N = 7,127)	Subtype 1 (N = 1,417)	Subtype 2 (N = 1,366)	Subtype 3 (N = 1,508)	Subtype 4 (N = 1,441)	Subtype 5 (N = 1,395)	P value
Serum sodium (mmol/L)							
0–24 h	140.22 (5.49)	140.74 (4.84)	139.21 (5.01)	140.34 (4.41)	137.68 (5.42)	143.17 (6.15)	<0.001
24–48 h	139.87 (5.50)	139.80 (5.26)	139.85 (5.83)	140.13 (5.22)	139.64 (5.63)	139.94 (5.56)	0.175
48–72 h	140.09 (5.69)	139.66 (5.20)	140.11 (6.00)	140.37 (5.12)	142.33 (6.07)	137.88 (5.11)	<0.001
72–96 h	140.57 (5.78)	140.24 (4.82)	140.44 (5.63)	140.47 (4.62)	144.51 (6.26)	137.09 (4.94)	<0.001
UO (mL)							
0–24 h	105.00 [50.00, 200.00]	100.00 [45.00, 156.00]	205.00 [125.00, 300.00]	75.00 [40.00, 125.00]	100.00 [50.00, 180.00]	103.00 [45.00, 200.00]	<0.001
24–48 h	190.00 [110.00, 300.00]	150.00 [90.00, 221.00]	180.00 [100.00, 290.00]	240.00 [165.00, 350.00]	215.00 [135.00, 330.00]	170.00 [85.00, 280.00]	<0.001

Characteristics	Total (N = 7,127)	Subtype 1 (N = 1,417)	Subtype 2 (N = 1,366)	Subtype 3 (N = 1,508)	Subtype 4 (N = 1,441)	Subtype 5 (N = 1,395)	P value
48–72 h	215.00 [130.00, 335.00]	220.00 [145.00, 325.00]	145.00 [70.25, 229.75]	295.00 [200.00, 435.00]	240.00 [160.00, 360.00]	185.00 [86.00, 300.00]	<0.001
72–96 h	240.00 [140.00, 375.00]	390.00 [260.00, 555.00]	155.00 [75.00, 250.00]	235.00 [155.00, 330.00]	250.00 [165.00, 390.00]	200.00 [100.00, 320.00]	<0.001

ICU – intensive care unit, UO – urine output.

Table S4. Log-rank test results for 30-day survival across subtypes.

**Comparison**	Log-rank P value
**30-day mortality**	
Subtype 1 vs Subtype 2	<0.001
Subtype 1 vs Subtype 3	0.154
Subtype 1 vs Subtype 4	<0.001
Subtype 1 vs Subtype 5	<0.001
Subtype 2 vs Subtype 3	<0.001
Subtype 2 vs Subtype 4	<0.001
Subtype 2 vs Subtype 5	0.070
Subtype 3 vs Subtype 4	0.031
Subtype 3 vs Subtype 5	<0.001
Subtype 4 vs Subtype 5	0.057

Table S5. Association between sepsis subtypes and secondary outcomes.

**Characteristic**	**HR (95% CI), P value**
	**Model 2**
90-day mortality	
Subtype 1	Ref.
Subtype 2	1.843 (1.566–2.169), <0.001
Subtype 3	1.218 (1.027–1.444), 0.024
Subtype 4	1.296 (1.094–1.535), 0.003
Subtype 5	1.490 (1.263–1.758), <0.001
In-hospital mortality	
Subtype 1	Ref.
Subtype 2	1.834 (1.559–2.157), <0.001
Subtype 3	1.211 (1.022–1.435), 0.027
Subtype 4	1.285 (1.085–1.522), 0.004
Subtype 5	1.480 (1.255–1.746), <0.001

Model 2: adjusted for age, race, sex, BMI, sepsis onset timing (pre-ICU/post-ICU), heart rate, SpO_2, lactate, anion gap, chloride, potassium, platelet count, pH, blood urea nitrogen, white blood cell count, baseline estimation of glomerular filtration rate, fluid balance, calcium, invasive ventilation, β-blockers, vasoactive drugs, mannitol treatment, diuretic therapy, renal replacement therapy, albumin infusion, GCS, SAPS II, SOFA score, congestive heart failure, chronic pulmonary disease, acute liver failure, renal disease, diabetes, hypertension.
BMI, body mass index; SpO_2, peripheral oxygen saturation; pH, potential of hydrogen; GCS, Glasgow Coma Scale; SAPS II, Simplified Acute Physiology Score II; SOFA – Sequential Organ Failure Assessment.

Table S6. Stability of trajectory classification under MNAR and MAR assumptions.

Comparison	Adjusted Rand index (ARI)	Number with changed trajectory assignment	Proportion changed
Moderate MNAR vs MAR	0.950	147 / 7,127	2.06%
Severe MNAR vs MAR	0.925	220 / 7,127	3.09%

ARI – adjusted Rand index, MAR – missing at random, MNAR – missing not at random. The number and proportion of patients with changed trajectory classification were calculated relative to the MAR scenario.

Table S7. Main Cox hazard ratios and percentage changes under MAR and MNAR assumptions.

Comparison	MAR HR	Moderate MNAR HR	Moderate change	Severe MNAR HR	Severe change
Subtype 2 vs Subtype 1	1.748	1.763	+0.89%	1.756	+0.44%
Subtype 3 vs Subtype 1	1.201	1.194	-0.63%	1.188	-1.09%
Subtype 4 vs Subtype 1	1.323	1.337	+1.13%	1.353	+2.32%
Subtype 5 vs Subtype 1	1.440	1.441	+0.09%	1.400	-2.74%
Maximum absolute change	-	-	1.13%	-	2.74%

HRs were estimated using the Cox proportional hazards model. Percentage change was calculated relative to the MAR scenario. HR – hazard ratio, MAR – missing at random, MNAR – missing not at random.

Table S8. Association between trajectory subtypes and 30-day mortality in the mean-exposure sensitivity analysis.

Trajectory subtype	**HR (95% CI), P value**
Subtype 1	**Ref.**
Subtype 2	1.318 (1.006–1.726), 0.045
Subtype 3	1.395 (0.992–1.963), 0.056
Subtype 4	1.673 (1.381–2.027), <0.001
Subtype 5	1.783 (1.468–2.165), <0.001

In this sensitivity analysis, mean values within each 24-hour time window were used to define serum sodium and urine output exposures. Subtype 1 was used as the reference group. CI – confidence interval, HR – hazard ratio.

Table S9. Cross-tabulation of K = 4 and K = 5 trajectory classifications.

K = 4 \ K = 5	Subtype 1	Subtype 2	Subtype 3	Subtype 4	Subtype 5
Subtype 1	14	1,215	870	12	93
Subtype 2	9	145	102	1,351	0
Subtype 3	1,268	6	448	78	20
Subtype 4	126	0	88	0	1,282

Rows represent trajectory subtypes from the K = 4 model; columns represent trajectory subtypes from the K = 5 model. Values are patient counts.

Table S10. Model diagnostic metrics for K = 5 and K = 6/K = 7 models after relaxing the 10% class-size criterion.

**Model**	**Minimum class proportion**	**Number of classes <10%**	**Minimum average posterior probability**	**BIC**	**Range of 30-day mortality**
K = 5	19.2%	0	0.83	123,813	16.2%–27.3%
K = 6	10.0%	0	0.72	123,954	16.2%–27.6%
K = 7	8.1%	1	0.69	122,803	15.9%–28.1%

The <10% class count indicates the number of trajectory classes with a sample proportion below 10%. BIC, Bayesian Information Criterion.

Table S11. Association between trajectory subtypes and 30-day mortality in the extended cohort with ICU length of stay ≥ 48 h.

Trajectory subtype	**HR (95% CI), P value**
Subtype 1	**Ref.**
Subtype 2	1.839 (1.628–1.994), <0.001
Subtype 3	1.730 (1.550–1.921), <0.001
Subtype 4	1.660 (1.460–1.902), <0.001
Subtype 5	1.201 (0.870–1.430), 0.076

This sensitivity analysis was conducted in the extended cohort of patients with ICU length of stay ≥ 48 h. Subtype 1 was used as the reference group. CI – confidence interval, HR – hazard ratio, ICU – intensive care unit.

Table S12. Association between trajectory subtypes and 30-day mortality in the LASSO-selected parsimonious Cox model.

**Variable**	**HR (95% CI)**	**P value**
Subtype 1	Ref.	Ref.
Subtype 2	1.840 (1.555–2.176)	<0.001
Subtype 3	1.218 (1.022–1.453)	0.028
Subtype 4	1.307 (1.098–1.556)	0.003

Subtype 5	1.548 (1.306–1.835)	<0.001

Subtype 1 was used as the reference group. The parsimonious Cox model was constructed using covariates selected by LASSO regression. CI – confidence interval, HR – hazard ratio, LASSO – least absolute shrinkage and selection operator.

Table S13. Comparison of trajectory-subtype hazard ratios for 30-day mortality between the original Cox and time-varying Cox models.

**Comparison**	**Original Cox**	**Time-varying Cox**
Subtype 2 vs Subtype 1	1.850 (1.563–2.189), <0.001	1.490 (1.250–1.781), <0.001
Subtype 3 vs Subtype 1	1.222 (1.025–1.458), 0.026	1.151 (0.960–1.370), 0.102
Subtype 4 vs Subtype 1	1.309 (1.099–1.558), 0.003	1.142 (0.940–1.372), 0.257
Subtype 5 vs Subtype 1	1.530 (1.290–1.815), <0.001	1.270 (1.061–1.524), 0.023

Subtype 1 was used as the reference group. The time-varying Cox model incorporated repeated measures across the four time windows. CI – confidence interval, HR – hazard ratio.

Table S14. Association of time-varying dynamic exposures with 30-day mortality.

**Dynamic exposure**	**HR (95% CI)**	**P value**
**Serum sodium**	1.120 (1.040–1.211)	**0.004**
Urine output	0.681 (0.624–0.746)	<0.001
Hyponatraemia	1.483 (1.273–1.731)	<0.001
Hypernatraemia	1.564 (1.345–1.820)	<0.001

Dynamic exposures were incorporated into the time-varying Cox model. Hyponatraemia and hypernatraemia were defined according to the prespecified serum sodium thresholds used in the analysis. CI – confidence interval, HR – hazard ratio.

Table S15. 30-day mortality and SOFA score in the original five trajectory subtypes.

Trajectory subtype	**n (%)**	**30-day mortality**	**Mean SOFA score**
Subtype 1	1,417 (19.9%)	16.2%	6.65
Subtype 2	1,366 (19.2%)	27.3%	6.16
Subtype 3	1,508 (21.2%)	18.3%	6.25
Subtype 4	1,441 (20.2%)	21.4%	6.60
Subtype 5	1,395 (19.6%)	24.3%	7.08

Percentages indicate the proportion of patients in each trajectory subtype. SOFA – Sequential Organ Failure Assessment.

Table S16. Internal validation and bootstrap Cox model stability.

**Validation analysis**	**Metric/comparison**	**Result**
50 repeated 70/30 split validations	Accuracy	0.759 (95% CI: 0.742–0.773)
50 repeated 70/30 split validations	ARI	0.493 (95% CI: 0.463–0.515)
100 bootstrap Cox resamples	Subtype 2 vs Subtype 1	HR = 1.850 (95% CI: 1.582–2.171)
100 bootstrap Cox resamples	Subtype 3 vs Subtype 1	HR = 1.240 (95% CI: 1.040–

		1.430)
100 bootstrap Cox resamples	Subtype 4 vs Subtype 1	HR = 1.321 (95% CI: 1.124–1.532)
100 bootstrap Cox resamples	Subtype 5 vs Subtype 1	HR = 1.575 (95% CI: 1.313–1.835)

Accuracy and ARI were calculated from 50 repeated 70/30 split validations. Bootstrap Cox estimates were obtained from 100 resampling iterations. ARI – adjusted Rand index, CI – confidence interval, HR – hazard ratio.

Table S17. Transition between early 48-hour and full 0–96-hour trajectory risk states and 30-day mortality.

Transition group	Sample size (n)	Percentage (%)	Deaths (n)	30-day mortality (%)	HR (95% CI)	P value
Persistently low/moderate risk	2,465	34.59	428	17.36	Ref.	Ref.
Improved (early high risk to final low/moderate risk)	460	6.45	78	16.96	0.94 (0.74–1.20)	0.617
Worsened (early low/moderate risk to final high risk)	2,501	35.09	569	22.75	1.29 (1.14–1.47)	<0.001
Persistently high risk	1,701	23.87	452	26.57	1.59 (1.39–1.82)	<0.001

Early risk status was defined using trajectories based on the first 48 hours of data, whereas final risk status was defined using the full 0–96-hour trajectory model. The persistently low/moderate-risk group was used as the reference group. CI – confidence interval, HR – hazard ratio.

Table S18. Subgroup analysis among patients with early high-risk status.

Comparison	HR (95% CI)	P value
Improved group	Ref.	Ref.
Persistently high-risk group	1.73 (1.36–2.21)	<0.001

This analysis was restricted to patients classified as high risk during the first 48 h. The improved group was used as the reference group. CI – confidence interval, HR – hazard ratio.
